# Global mechanical power does not determine regional energy distribution in heterogeneous lungs

**DOI:** 10.3389/fphys.2026.1867485

**Published:** 2026-07-08

**Authors:** Tomasz Urbankowski, Emilia Urbankowska, Marek Darowski

**Affiliations:** 1 Department of Modeling and Supporting of Internal Organs Functions, Nalecz Institute of Biocybernetics and Biomedical Engineering, Polish Academy of Sciences, Warsaw, Poland; 2 Independent Researcher, Warsaw, Poland

**Keywords:** energy partitioning, lung heterogeneity, mechanical power, two-compartment model, ventilator-induced lung injury

## Abstract

**Introduction:**

Mechanical power (MP) integrates key determinants of ventilatory load and has been associated with ventilator-induced lung injury (VILI). However, because MP is typically derived from airway-opening measurements, it represents a global metric that may not reflect how energy is distributed within mechanically heterogeneous lungs. We aimed to investigate whether global MP uniquely determines regional energy partitioning under heterogeneous conditions.

**Methods:**

We conducted a deterministic in-silico study using a parallel two-compartment resistance–compliance model under volume-controlled ventilation. Systematic parameter sweeps over compliance and resistance ratios were performed, and a partition index (PI) was introduced to quantify preferential inspiratory energy delivery to the more compliant compartment.

**Results:**

Across the heterogeneity space, PI varied widely (0.352–0.800), while global MP ranged from 1.46 to 5.85 J/min. Importantly, iso-lines of MP overlapped regions with markedly different PI values, demonstrating that similar global MP can correspond to substantially different energy distributions. Among parameter sets matched within ±5% MP, the maximal difference in PI reached 0.39, indicating pronounced variability in preferential energetic loading despite comparable global energy delivery.

**Discussion:**

These findings demonstrate a structural non-identifiability: global mechanical power does not uniquely determine how inspiratory energy is partitioned in heterogeneous lungs. The proposed PI provides a compact descriptor of impedance-driven energetic routing and highlights conditions in which acceptable global MP may mask disproportionate regional energy exposure. Incorporating heterogeneity-aware information may therefore be necessary to interpret MP and guide safer ventilation strategies.

## 1 Introduction

Mechanical power (MP) represents the rate at which a ventilator transfers energy to the respiratory system and integrates key determinants of ventilator load (tidal volume, pressure amplitude/driving pressure, inspiratory flow, respiratory rate, and positive end-expiratory pressure (PEEP)-related components) (Gattinoni et al., 2016). A recent systematic review in adult invasively ventilated patients supports an association between higher global MP and ventilator-induced lung injury (VILI)-related outcomes, reinforcing MP as a clinically relevant risk marker (Urbankowski et al., 2025). Because MP is typically estimated from airway-opening measurements, it is a global metric. However, MP remains a surrogate for injurious energy transfer and cannot, by itself, capture the spatial distribution of stress/strain or the dissipation pathways that ultimately determine VILI risk (Bates et al., 2024).

In mechanically heterogeneous lungs, therefore, global measures may fail to reflect regional stress exposure. A single set of ventilator settings can preferentially route flow and energy toward pathways that offer the lowest mechanical opposition to inflation. In respiratory mechanics, this opposition is often described as impedance and reflects the combined effects of airway resistance and elastic load, the latter being determined by elastance, i.e., the inverse of compliance. Thus, depending on resistance, elastance, and regional time constants, some lung units may receive a disproportionate share of flow and inspiratory energy, potentially creating preferential energetic loading in more compliant regions despite an apparently acceptable global MP (Mead et al., 1970; Cressoni et al., 2014; Marini et al., 2020). Such heterogeneity is not an edge case—pronounced inhomogeneity is a defining feature of acute respiratory distress syndrome (ARDS) and is frequently accentuated by clinically common phenotypes, including focal or lobar consolidation, atelectasis/derecruitment, unilateral disease with left–right asymmetry (e.g., pneumonia, aspiration, post-operative or trauma-related patterns), and mixed resistance–compliance abnormalities (Mead et al., 1970; Cressoni et al., 2014; Berg et al., 2019). In these settings, a compact indicator of impedance-driven “preferential routing” could serve as a phenotype flag—a signal that acceptable global MP may still coincide with disproportionate energetic exposure of the more ventilated pathway. A mechanistic framework that quantifies how inspiratory energy is partitioned across lung units can therefore clarify when global MP underestimates regional energetic burden and provide a bridge between global ventilator metrics and heterogeneity-aware ventilation strategies. In-silico experimentation and “virtual patient” modeling are increasingly used in biomedical research and education, offering a controllable framework to probe cardiopulmonary phenomena when *in-vivo* experimentation is limited (Stankiewicz et al., 2017; Zieliński et al., 2022).

Here, we use a deterministic in-silico model to examine energy partitioning in a heterogeneous system. We propose a simple partition index (PI) to quantify preferential energetic loading toward the more compliant compartment and test whether heterogeneity can decouple PI from global MP.

## 2 Materials and methods

### 2.1 Study design

Deterministic in-silico simulation study using a parallel two-compartment respiratory system model under volume-controlled ventilation (VCV) with systematic parameter sweeps.

### 2.2 Model structure

**Figure 1:**
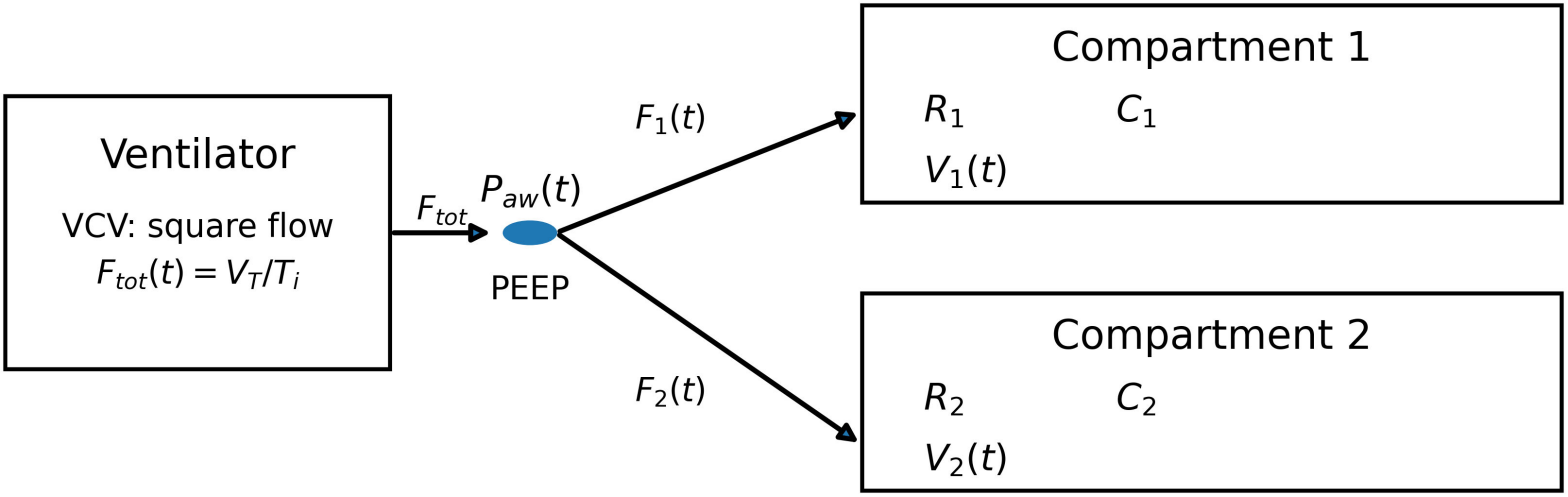
Schematic of the two-compartment R–C model. A volume-controlled ventilator delivers a constant inspiratory flow $F_{\text{tot}}\left(t\right)=V_{T}/T_{i}$ (square flow profile) to a shared airway opening with pressure *P_aw_*(*t*). The flow splits into compartmental flows *F*_1_(*t*) and *F*_2_(*t*) entering two parallel R–C units characterized by resistances *R*_1_, *R*_2_, compliances *C*_1_, *C*_2_, and resulting compartment volumes *V*_1_(*t*) and *V*_2_(*t*). Positive end-expiratory pressure (PEEP) is applied at the airway opening. The blue ellipse denotes the shared airway opening (junction) where pressure *P_aw_*(*t*) is defined and flow splits.


A two-compartment parallel model was implemented, representing two lung regions exposed to a shared airway opening pressure *P_aw_*(*t*) and common PEEP, as shown in **Figure 1**. PEEP was treated as the end-expiratory reference pressure applied at the airway opening. Therefore, the dynamic pressure driving inspiratory flow was expressed relative to PEEP as:

$$\Delta P_{aw}\left(t\right)=P_{aw}\left(t\right)-PEEP$$

For each compartment i, *V_i_*(*t*) denotes the compartmental volume above the end-expiratory equilibrium volume at PEEP, and *F_i_*(*t*) denotes compartmental flow, defined as positive into the lung. The compartmental pressure–volume–flow relation was therefore written in incremental form as:


$$\Delta P_{aw}\left(t\right)=\frac{V_{i}\left(t\right)}{C_{i}}+R_{i}F_{i}\left(t\right)$$

or equivalently:


$$P_{aw}\left(t\right)=PEEP+\frac{V_{i}\left(t\right)}{C_{i}}+R_{i}F_{i}\left(t\right)$$

This formulation does not imply that PEEP is an additional inspiratory driving pressure; rather, PEEP defines the baseline pressure from which incremental pressure, volume, and energy are measured.

During inspiration under volume-controlled ventilation (VCV), the prescribed total airway-opening flow, F_tot_(t), satisfies the parallel constraint:


$$F_{tot}\left(t\right)=F_{1}\left(t\right)+F_{2}\left(t\right)$$

and both compartments share the same airway opening pressure *P_aw_*(*t*). During expiration, the airway opening pressure is clamped to the set PEEP, and compartmental flows are computed passively from the resistance-compliance (RC) dynamics without enforcing a prescribed total-flow constraint at each expiratory time step. Accordingly, the airway-opening flow is:


$$F_{tot}\left(t\right)=F_{1}\left(t\right)+F_{2}\left(t\right)$$

which is generally non-zero during expiration and approaches 0 only at end-expiratory equilibrium.

During inspiration, with prescribed *F_tot_*(*t*), *P_aw_*(*t*) was obtained at each time step from the closed-form expression derived from the incremental pressure formulation (see Appendix A for full derivation). The compartmental flows were computed as:


$$F_{i}\left(t\right)=\frac{P_{aw}\left(t\right)-PEEP-V_{i}\left(t\right)/C_{i}}{R_{i}}$$

or, equivalently:


$$F_{i}\left(t\right)=\frac{\Delta P_{aw}\left(t\right)-V_{i}\left(t\right)/C_{i}}{R_{i}}$$

The compartmental volumes were then updated by forward Euler integration:


$$V_{i}\left(t+\Delta t\right)=V_{i}\left(t\right)+F_{i}\left(t\right)\Delta t$$

Expiration was modeled as a passive phase under:


$$P_{aw}\left(t\right)=\text{PEEP}$$

and therefore:


$$\Delta P_{aw}\left(t\right)=0$$

Each compartment emptied according to its RC dynamics:


$$F_{i}\left(t\right)=-\frac{V_{i}\left(t\right)}{R_{i}C_{i}}$$

Accordingly, the total expiratory flow at the airway opening:


$$F_{tot}\left(t\right)=F_{1}\left(t\right)+F_{2}\left(t\right)$$

is naturally non-zero during most of expiration and approaches zero only at end-expiratory equilibrium. Expiration was simulated only to close the cycle and set initial conditions for the subsequent breath; all reported energy and mechanical power metrics are computed over inspiration only.

### 2.3 Model assumptions

Each compartment was modeled as a linear RC element (linear compliance and linear resistance). Compartments interacted only through the shared airway pressure and the flow constraint. The model intentionally excluded inertance, nonlinear compliance, recruitment/derecruitment, viscoelasticity, and pendelluft, to isolate the effects of resistance–compliance heterogeneity on inspiratory energy partitioning.

### 2.4 Ventilator waveform and numerical integration

The primary mode was volume-controlled ventilation (VCV) with a square (constant) inspiratory flow waveform. For a given tidal volume (VT) and inspiratory time (T_i_), the commanded total inspiratory flow was prescribed as


$$F_{tot}\left(t\right)=\frac{VT}{T_{i}},0\leq t\leq T_{i}.$$

During expiration, no prescribed total-flow waveform was imposed and expiration was passive with the airway opening pressure clamped at


$$P_{aw}\left(t\right)=PEEP.$$

The respiratory period was set by the respiratory rate (RR). Simulations used a fixed time step dt=0.001 s, chosen as a practical compromise between numerical accuracy and computation time for the 61×61 parameter sweep.

### 2.5 Initial conditions

At simulation start, compartmental volumes were initialized at end-expiratory equilibrium relative to PEEP (i.e., V_i_ (0) = 0 referenced to PEEP), and the subsequent dynamics were simulated over full breathing cycles with fixed settings.

### 2.6 Pressure solution and state update

During inspiration, with prescribed F_tot_(t), P_aw_(t) was obtained at each time step from the closed-form expression (see Appendix A for full derivation). Compartmental flows were then computed as


$$F_{i}\left(t\right)=\frac{P_{aw}\left(t\right)-PEEP-\frac{V_{i}\left(t\right)}{C_{i}}}{R_{i}},$$

and compartmental volumes were updated by forward (Euler) integration:


$$V_{i}\left(t+dt\right)=V_{i}\left(t\right)+F_{i}\left(t\right)\;dt.$$

Expiration was modeled as a passive phase under P_aw_ (t) = PEEP, and each compartment emptied according to its RC dynamics:


$$F_{i}\left(t\right)=\frac{P_{aw}\left(t\right)-PEEP-\frac{V_{i}\left(t\right)}{C_{i}}}{R_{i}}=-\frac{V_{i}\left(t\right)}{C_{i}R_{i}},\text{with}\frac{dV_{i}}{dt}=F_{i}.$$

Accordingly, the total expiratory flow at the airway opening,


$$F_{exp}\left(t\right)=F_{1}\left(t\right)+F_{2}\left(t\right),$$

is naturally non-zero during most of expiration and approaches zero only at end-expiratory equilibrium. Expiration was simulated only to close the cycle and set initial conditions for the subsequent breath; all reported energy and mechanical power metrics are computed over inspiration only.

Baseline settings for representative figures and the primary heterogeneity sweep were VT = 0.45 L, T_i_ = 1.0 s, RR = 18/min, and PEEP = 8 cmH_2_O, with baseline compartment 1 mechanics C_1_ = 0.04 L/cmH_2_O and R_1_ = 8 cmH_2_O · s/L.

### 2.7 Energy, mechanical power, and partition index

Inspiratory energy delivered to each compartment was computed over the inspiratory phase as the time integral of incremental (above-PEEP) airway-opening pressure–flow power:


$$E_{i,\text{inc}}=\int_{0}^{T_{I}}(P_{aw}\left(t\right)-\text{PEEP})\;F_{i}\left(t\right)\;dt,i=1,2.$$

Because P_aw_ is expressed in cmH_2_O and volume in liters, energies were converted to joules using 1 cmH_2_O·L = 0.098 J. All reported results use the incremental (above-PEEP) definition.

### 2.8 Interpretation of compartmental energy

In this parallel model, the inspiratory incremental (above-PEEP) energy delivered to compartment *i* is defined as


$$E_{i,\text{inc}}=\int_{0}^{T_{I}}(P_{aw}\left(t\right)-PEEP)\;F_{i}\left(t\right)\;dt,$$

and represents airway-opening delivered energy apportioned by compartmental flow (i.e., flow-apportioned energy). Because the same airway-opening pressure acts on both compartments, E_i, inc_ quantifies how the ventilator’s delivered energy is partitioned between parallel pathways as a function of their impedance. Importantly, E_i, inc_ is not a direct estimate of regional parenchymal stress/strain energy; it aggregates energy associated with both resistive dissipation and elastic storage along the pathway represented by compartment i. Accordingly, E_i, inc_ is used here as a proxy metric of preferential energetic loading driven by flow partitioning in heterogeneous parallel mechanics. Unless otherwise stated, all energies E_i, inc_ and MP_total_ refer to the incremental (above-PEEP) form.

Total inspiratory energy per breath was computed as the sum of compartmental incremental energies (above PEEP):


$$E_{total}=E_{1,\text{inc}}+E_{2,\text{inc}}.$$

Global mechanical power was then defined as


$$MP_{total}=E_{total}\cdot RR,$$

where RR is the respiratory rate (breaths/min).

The partition index (PI) quantified preferential energetic loading as the fraction of total inspiratory energy delivered to the more compliant compartment:


$$PI=\frac{E_{\text{more-compliant},\text{inc}}}{E_{total}}.$$

Accordingly, PI is interpreted as a partitioning proxy (i.e., how energy is distributed between compartments) rather than a direct measure of regional tissue energy. To orient the index toward the potentially more strain-prone pathway in terms of preferential flow routing, we define the numerator using the more compliant compartment by convention. Specifically, E_more-compliant, inc_ is the incremental inspiratory energy delivered to the compartment with higher compliance (C_more_ = max (C_1_, C_2_); if C_1_ = C_2_, we set E_more-compliant, inc_ = E_1, inc_ by convention. Under symmetric mechanics (C_1_ = C_2_ and R_1_ = R_2_), this yields PI ≈ 0.5. For reference, a symmetric alternative is


$$PI_{max}=\frac{\text{max}\left(E_{1,\text{inc}},E_{2,\text{inc}}\right)}{E_{total}},$$

which encodes preferential loading without conditioning on compliance ordering.

In the primary (incremental) definition, E_more-compliant, inc_ and E_total_ are computed from (P_aw_ – PEEP), so PI focuses on inspiratory pressure-amplitude allocation. If instead absolute pressure were used, $E_{i,\text{abs}}=\int_{0}^{T_{I}}P_{aw}\left(t\right)\;F_{i}\left(t\right)\;dt$, an additional static term PEEP would be introduced into each compartment’s inspiratory energy, which could shift PI slightly depending on resistance–compliance heterogeneity (via ΔV_i_). In all primary analyses we report PI and MP_total_ computed from incremental (above-PEEP) pressure (P_aw_ – PEEP) and over inspiration only, to exclude static PEEP-related volume allocation effects and to focus on the dynamic (inspiratory) component of delivered energy. Thus, reported values correspond to inspiratory (dynamic) mechanical power; static PEEP-related power is intentionally excluded.

### 2.9 Heterogeneity sweep and analyses

**Figure 2:**
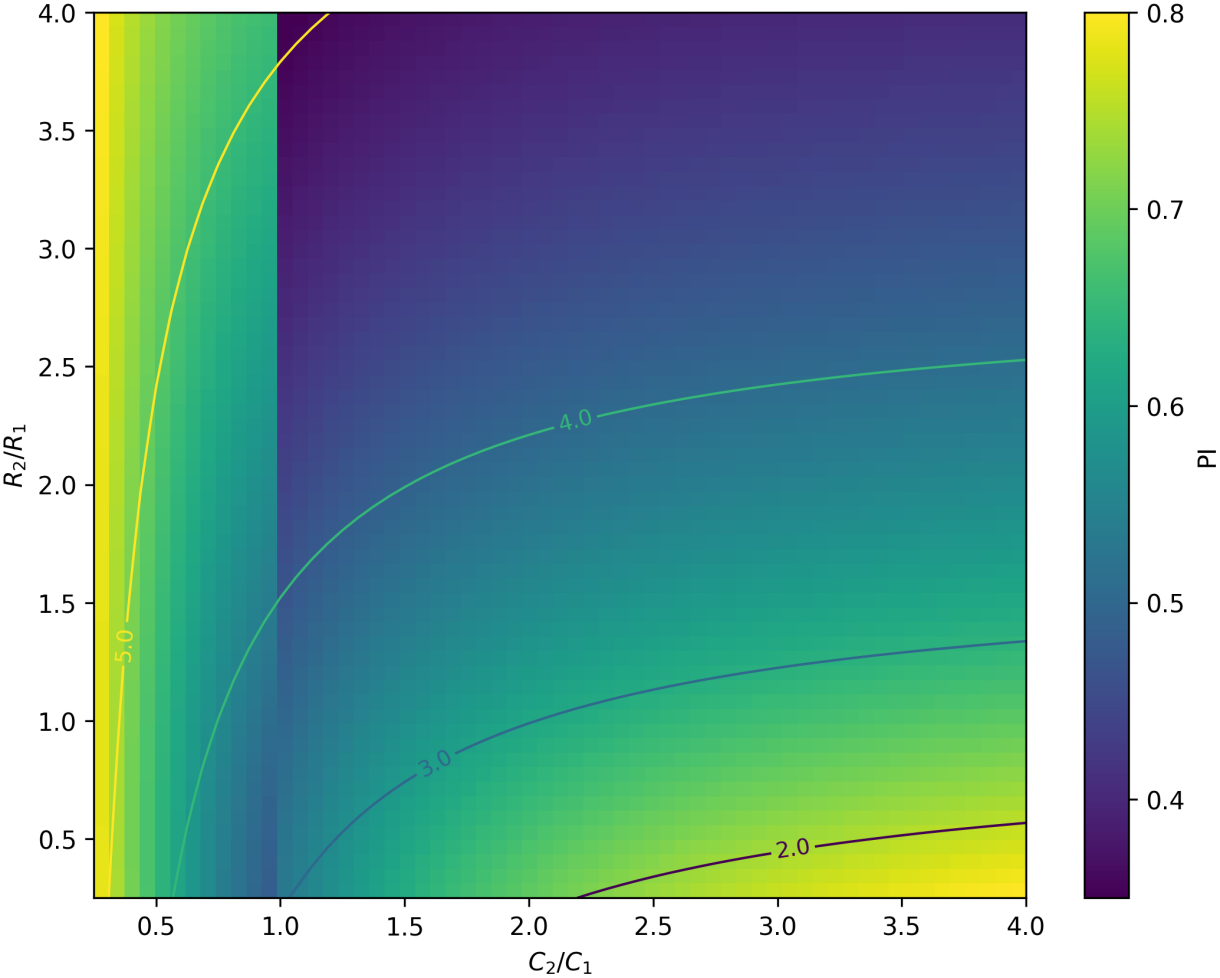
Partition index (PI) across compliance–resistance heterogeneity with iso-lines of global mechanical power. Heatmap showing the partition index (PI) across the heterogeneity plane defined by compliance and resistance ratios (*C*_2_/*C*_1_ on the x-axis; *R*_2_/*R*_1_ on the y-axis; both swept 0.25–4.0 on a 61×61 grid). PI is defined as the fraction of total inspiratory incremental (above-PEEP) energy delivered to the more compliant compartment: $PI=E_{\text{more-compliant}}/E_{\text{total}}$, where $E_{i}=\int_{0}^{T_{i}}(P_{aw}-PEEP)\;F_{i}\;dt$ and $E_{\text{total}}=E_{1}+E_{2}$. By convention, $E_{\text{more-compliant}}$ uses the compartment with higher compliance (if *C*_1_ = *C*_2_, compartment 1 is used). Contour lines denote iso-values of global inspiratory mechanical power $MP_{\text{total}}=E_{\text{total}}\cdot RR$ (levels: 2.0, 3.0, 4.0, and 5.0 J/min). Baseline settings: volume-controlled ventilation with square inspiratory flow (*V_T_* = 0.45 L, *T_i_* = 1.0 s, *RR* = 18/min, *PEEP* = 8 cmH_2_O), with compartment-1 mechanics fixed at *C*1 = 0.04 L/cmH_2_O and *R*_1_ = 8 cmH_2_O·s/L. Regions with similar *MP*_total_ can exhibit markedly different PI, illustrating that global mechanical power does not uniquely determine compartmental energy partitioning in heterogeneous parallel mechanics.

**Figure 3:**
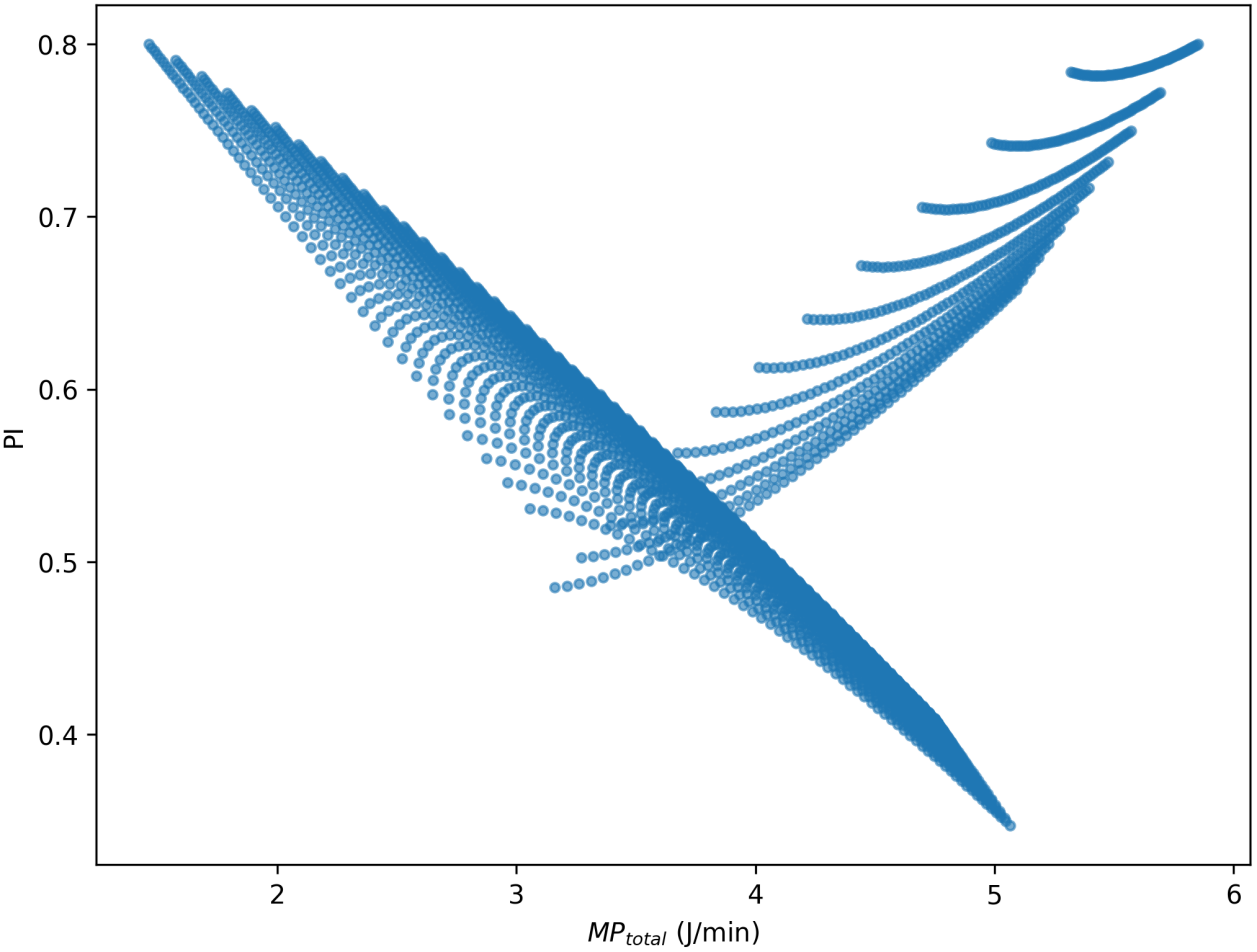
Partition index versus global mechanical power across the compliance–resistance heterogeneity sweep. Scatter plot of the partition index (PI) versus global inspiratory mechanical power (MP_total_) across the 61×61 sweep of heterogeneity ratios (C2/C1 and R2/R1, each 0.25–4.0) under baseline volume-controlled ventilation (VT = 0.45 L, Ti=1.0 s, RR = 18/min, PEEP = 8 cmH_2_O; C1 = 0.04 L/cmH_2_O, R1 = 8 cmH_2_O·s/L). PI is defined as the fraction of total inspiratory incremental (above-PEEP) energy delivered to the more compliant compartment, PI = E_more-compliant_/E_total_, with energies computed over inspiration as E_i, inc_ = ∫(Paw−PEEP)·Fi dt and MP_total_ = (E1_, inc+_E2_, inc_)·RR. Despite MP_total_ matching within ±5%, PI could still differ markedly; the maximal separation within this matching window was ΔPI=0.39, illustrating that similar global mechanical power can coincide with substantially different preferential energetic loading.

To map the relationship between heterogeneity, the partition index (PI), and global mechanical power, we performed a grid sweep over compliance and resistance ratios (C_2_/C_1_ and R_2_/R_1_), each spanning 0.25–4.0, while holding compartment 1 mechanics constant (C_1_ = 0.04 L/cmH_2_O, R_1_ = 8 cmH_2_O·s/L). The parameter space was sampled on a uniform 61×61 grid (61 values per axis; step 0.0625). For each grid point, we computed PI, MP_total_, and compartmental incremental inspiratory energies E_1_, inc and E_2_, inc, and visualized PI as a heatmap with iso-MP_total_ contours (**Figure 2**). We further summarized the non-unique mapping between global mechanical power and partitioning by plotting PI versus MP_total_ across all 61×61 heterogeneity scenarios and quantifying the maximal PI separation observed among cases with matched MP_total_ within ±5% (**Figure 3**).

### 2.10 Software and reproducibility

The model, parameter sweeps, and analyses were implemented in Python (v3.9.6). Data handling used NumPy and pandas, and all figures were generated with Matplotlib using dedicated scripts. Simulations were executed locally on a macOS workstation (Python build: Clang 17.0.0). Numerical stability of the chosen time step (dt=0.001 s) was assessed by repeating a representative subset of simulations at 25 grid points (C2/C1 and R2/R1 ∈ {0.25, 0.5, 1, 2, 4}) using smaller time steps and treating dt=0.00025 s as the reference. The maximum relative deviation (vs dt=0.00025 s) was 0.040% for MP_total_ and 0.009% for PI at dt=0.0005 s, and 0.120% for MP_total_ and 0.028% for PI at dt=0.001 s, supporting dt=0.001 s as a practical compromise between accuracy and computation time for the full 61×61 sweep.

### 2.11 Ethical approval

This study did not involve human participants, animals, or identifiable human data, as it is based entirely on computational in-silico modeling. Accordingly, institutional review board approval and informed consent were not required. The study was conducted in accordance with applicable guidelines for research integrity.

## 3 Results

### 3.1 Representative waveforms demonstrate preferential loading and redistribution

**Figure 4:**
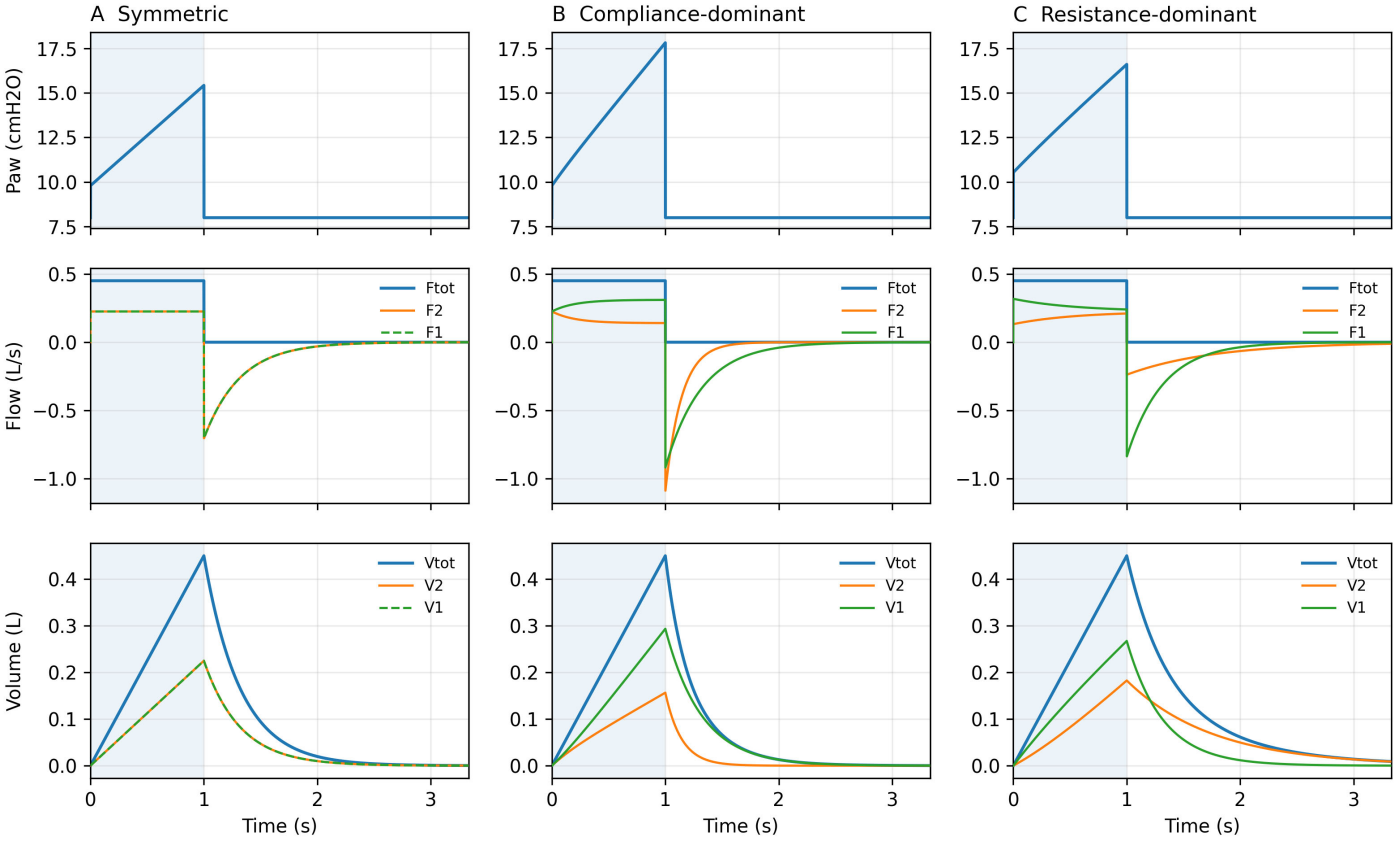
Representative waveforms illustrating redistribution under identical global settings. Airway opening pressure (Paw), flow, and volume waveforms for a single breath in a parallel two-compartment model under volume-controlled ventilation (VT = 0.45 L, Ti = 1.0 s, RR = 18/min, PEEP = 8 cmH_2_O). Baseline parameters were C1 = 0.04 L/cmH_2_O and R1 = 8 cmH_2_O·s/L. Panels show three illustrative heterogeneity scenarios: A, symmetric (C2/C1 = 1.00, R2/R1 = 1.00); B, compliance-dominant (C2/C1 = 0.45, R2/R1 = 1.00); and C, resistance-dominant (C2/C1 = 1.00, R2/R1 = 2.40). During inspiration, total flow satisfies Ftot(t) = F1(t) + F2(t), and compartmental volumes are obtained by time integration of flow. Waveforms illustrate how compliance versus resistance heterogeneity redistributes inspiratory flow and tidal volume between compartments despite identical ventilator settings and a shared Paw. Preferential energetic loading (PI) for the illustrated cases was 0.50 **(A)**, 0.67 **(B)**, and 0.58 **(C)**.

Under symmetric mechanics (C1=C2, R1=R2), inspiratory flow and volume were evenly distributed between compartments, yielding PI≈0.50 (**Figure 4A**). Compliance-dominant heterogeneity (C1>C2 with similar resistances) shifted inspiratory flow toward the more compliant compartment and increased PI (PI≈0.67 in a representative case; **Figure 4B**). Resistance-dominant heterogeneity (R1<R2 with similar compliances) preferentially routed early inspiratory flow toward the lower-resistance compartment and increased PI (PI≈0.58; **Figure 4C**). These waveforms illustrate that heterogeneity substantially alters compartmental flow and volume trajectories despite identical global ventilator settings and a shared P_aw_.

To triangulate model behavior against physiological intuition, we verified that PI approaches 0.5 under symmetric mechanics; increases as compliance disparity grows when resistances are matched; and shifts toward the lower-resistance pathway when compliances are matched—consistent with classic time-constant–governed flow partitioning in parallel RC systems.

### 3.2 PI varies nonlinearly across compliance–resistance heterogeneity

Across the compliance–resistance sweep, PI spanned a wide range (0.352–0.800; 5th–95th percentile: 0.400–0.745). Over the same sweep, MPtotal ranged from 1.46 to 5.85 J/min (5th–95th percentile: 2.21–5.20) and varied nonlinearly over the parameter space, with broad regions exhibiting strong preferential loading of the more compliant compartment (**Figure 2**). Iso-lines of MPtotal overlapped regions with markedly different PI values, indicating that global mechanical power does not uniquely determine how inspiratory energy is distributed between heterogeneous compartments.

### 3.3 Similar MP can coincide with markedly different partitioning

When parameter sets were matched within ±5% MP_total_, PI could still differ substantially (**Figure 3**). The maximal PI separation within this matching window was ΔPI=0.39, demonstrating that comparable global energy delivery can still coincide with meaningfully different preferential compartmental loading. Such MP-matched, higher-ΔPI pairs clustered along trade-off bands in the (C2/C1, R2/R1) plane, where resistance and compliance heterogeneity offset each other in MP_total_ but not in energy partitioning (PI).

## 4 Discussion

This in-silico study demonstrates that compliance and resistance heterogeneity can decouple global mechanical power from compartmental energy delivery. Even with fixed ventilator settings and similar MP_total_, differences in impedance reroute flow and volume toward the path of least impedance, increasing preferential inspiratory energy loading of the more compliant compartment.

This preferential partitioning can be described as preferential energetic loading. In this study, the term refers to airway-opening inspiratory energy partitioned according to compartmental flow (the time integral of (P_aw_−PEEP)·Fi), rather than a direct estimate of regional parenchymal stress/strain or tissue-level energy deposition. This is intentional: by aggregating both resistive dissipation and elastic storage along each pathway, the metric captures impedance-driven routing of ventilator-delivered energy at the airway opening.

The injury relevance of PI must therefore be interpreted cautiously. In the present model, PI is not a measure of injurious tissue-level energy dissipation. It is calculated from the time integral of above-PEEP airway-opening pressure multiplied by compartmental flow and therefore represents a pathway-level routing descriptor. As such, it combines recoverable elastic storage and airway resistive dissipation, while omitting recruitment/derecruitment, tissue viscoelasticity, nonlinear compliance, regional transpulmonary pressure, and parenchymal stress/strain.

This distinction is important in light of recent energy-based analyses of VILI. Bates et al. emphasized that purely elastic work is recoverable and is not injurious per se, except insofar as it correlates with overdistension, and that airway resistive dissipation is unlikely to cause clinically meaningful injury because its power intensity within tissues is low (Bates et al., 2024). Similarly, Gaver et al. quantified and partitioned dissipated mechanical energy into airflow-, tissue/surfactant-, and recruitment/derecruitment-related components in a porcine ARDS model (Gaver et al., 2025). They found that recruitment/derecruitment dissipation, despite accounting for only 2–5% of total dissipated energy, was the only measured component whose rank order and dose–response relationship aligned with physiological recovery assessed by oxygenation and respiratory-system compliance, whereas the larger airflow and tissue/surfactant components did not. These findings indicate that the biological relevance of mechanical energy depends not only on its magnitude, but also on where, how rapidly, and through which physical mechanism it is dissipated.

Accordingly, the present study should not be read as showing that preferential routing of elastic or airway resistive energy is itself injurious. Rather, it shows that global MP is structurally insufficient to determine regional energy routing in a heterogeneous system. Thus, in the absence of recruitment/derecruitment dynamics, preferential routing should be interpreted as a marker of mechanical heterogeneity and regional volume concentration, rather than as an independent injury mechanism. The possible relevance to VILI is indirect: preferential routing may mark conditions in which regional ventilation and volume delivery are concentrated in a subset of lung units, potentially increasing susceptibility to overdistension or interacting with recruitment/derecruitment if such nonlinear phenomena are present. These downstream mechanisms are not represented in the current model and require explicit modeling and experimental validation. Whereas Crooke et al. explicitly link compartmental intracycle power to modeled strain/strain-rate under multiple inspiratory flow profiles, our two-compartment analysis isolates the upstream question of energetic routing at the airway opening (PI) and demonstrates non-identifiability of regional energy partitioning from global MP even under a fixed VCV waveform (Crooke et al., 2022). Thus, PI is best regarded as a heterogeneity-aware routing metric and a hypothesis-generating phenotype flag, not as a validated regional VILI-risk index. Future extensions should explicitly incorporate recruitment/derecruitment, because this mechanism may determine whether preferential routing remains only a mechanical redistribution phenomenon or becomes linked to spatially focused injurious energy dissipation.

Importantly, the path of least impedance is not determined by compliance alone: flow partitioning depends on both resistance and compliance through the compartment time constant (tau = R·C), so early versus late inspiration may preferentially ventilate different units (Otis et al., 1956; Depta et al., 2025). This dynamic interaction is reflected in **Figure 2** by the strong dependence of PI on both compliance and resistance ratios—PI varies with R_2_/R_1_ even at fixed C_2_/C_1_—highlighting regions where compliance ratio alone is insufficient to predict partitioning. Accordingly, similar global MP values may correspond to very different energetic exposure scenarios at the compartment level. Our finding that similar global MP_total_ can coincide with markedly different compartmental energetic routing (PI) aligns with prior multi-compartment intracycle power modeling showing that compartmental deformation dynamics (strain and strain rate) do not necessarily track power delivery in a one-to-one manner and depend strongly on heterogeneity and flow profile (Crooke et al., 2022).

Simplified models are often criticized as being “too simple” to be clinically meaningful. In the present study, however, the two-compartment parallel R–C system is best understood as a minimal counterexample: the simplest architecture that (i) admits heterogeneity in compliance and resistance, (ii) enforces a shared airway-opening pressure, and (iii) allows dynamic flow redistribution under a prescribed global waveform. Demonstrating a decoupling between global mechanical power and energy partitioning in this minimal setting strengthens—rather than weakens—the central inference: the mapping from global MP to regional energetic exposure is not identifiable without information on heterogeneity. More complex multi-compartment, nonlinear, or recruitable models may refine the magnitude and topology of the PI surface, but they do not eliminate the fundamental non-uniqueness demonstrated here.

Clinically, these findings emphasize a limitation of airway-opening summary metrics: global MP may mask preferential energetic loading patterns that could be relevant to injury risk in phenotypes with pronounced heterogeneity or left–right asymmetry (Chi et al., 2020). More broadly, measurement and circuit-related factors can meaningfully bias delivered-volume/flow estimates, particularly when patient compliance is comparable to circuit compliance—highlighting why purely airway-opening, global quantities may diverge from the effective patient-level delivery in selected settings (Stankiewicz et al., 2020). The partition index provides a compact descriptor of preferential energetic routing and clarifies why heterogeneity information (imaging, regional ventilation distribution, or regional mechanics surrogates) may be required to interpret MP and rationalize ventilator adjustments.

PI is not yet a bedside-ready metric, but the concept of impedance-driven preferential routing can be approximated using clinically accessible surrogates of heterogeneity and regional ventilation distribution. The most pragmatic candidates are: (i) electrical impedance tomography (EIT)-derived regional tidal ventilation (Vt, region) and distribution shifts (Franchineau et al., 2024; Scaramuzzo et al., 2024), (ii) computed tomography (CT) or other imaging evidence of focal consolidation/atelectasis and left–right asymmetry (Gattinoni et al., 2001; Bouhemad et al., 2015), and (iii) time-constant imbalance surrogates inferred from flow–time behavior and/or EIT-derived regional filling/emptying dynamics (Lourens et al., 2000; Depta et al., 2025). In this framing, PI acts primarily as a phenotype/risk flag—signaling that an apparently acceptable global MP may still coincide with disproportionate energetic exposure of the more ventilated pathway.

Practically, the non-unique mapping shown in our sweep implies “similar global MP, different routing risk”, which can matter in common intensive care unit (ICU) scenarios: (1) during PEEP titration in focal/patchy ARDS, two PEEP levels may yield comparable MP yet very different regional ventilation allocation on EIT; (2) in unilateral pneumonia/aspiration with marked left–right asymmetry, global MP may appear acceptable while one lung receives most of the delivered energy; and (3) with waveform/flow adjustments under VCV, matched MP settings may still redistribute early versus late inspiratory delivery in the presence of time-constant heterogeneity. These examples do not claim validated bedside computation of regional mechanical power; rather, they motivate combining global MP with heterogeneity surrogates (EIT/CT/time constants) to identify when preferential routing may make global MP clinically misleading.

A pragmatic pathway toward such an approach is to use EIT primarily as a regional ventilation allocator: breath-by-breath estimates of regional tidal ventilation (Vt, region) and regional compliance proxies (e.g., impedance-derived ΔV, region per unit driving pressure) can be combined with airway pressure and flow to estimate an airway-opening, flow- or volume-apportioned fraction of delivered inspiratory energy (Franchineau et al., 2024; Scaramuzzo et al., 2024). Importantly, because EIT-derived Vt, region primarily reflects ventilation distribution without separating resistive versus elastic components–and without direct validation against regional stress/strain–it should be interpreted as a surrogate of impedance-driven routing rather than a measure of regional mechanical power. Recent validation against whole-lung 4D-CT in mechanically ventilated ICU patients showed that EIT-derived regional ventilation dynamics track CT-derived regional air-volume changes with high phase-consistent agreement across ventral–dorsal and left–right regions of interest (ROIs), except when the regional ventilation signal is extremely small (Rixner et al., 2025).

Similarly, pronounced left–right asymmetry in aeration on CT (or focal derecruitment/consolidation on lung ultrasound), together with differences in regional filling–emptying dynamics (time-constant behavior inferred from the expiratory flow–time curve or EIT-derived regional time constants), may help identify phenotypes prone to preferential energetic loading despite acceptable global MP (Lourens et al., 2000; Cressoni et al., 2014; Bouhemad et al., 2015; Karagiannidis et al., 2018; Scaramuzzo et al., 2024; Depta et al., 2025).

Relatedly, the proposed Power Compliance Index (mechanical power indexed to compliance) provides a pragmatic example of normalizing power to the mechanics (and thus effective “size”) of the ventilated unit, supporting heterogeneity-aware interpretation beyond global MP alone (Keitoku et al., 2022). Bench modeling of pendelluft further supports this concept: regional gas redistribution is primarily governed by interregional pressure gradients and mechanical heterogeneity, while adjustments in conventional ventilator settings exert only limited influence on the severity of the phenomenon (Enokidani et al., 2021).

Incorporating recruitment/derecruitment is particularly relevant because experimental evidence suggests that the injuriousness of recruitment-related dissipation stems from its spatial and temporal focusing (high power intensity), rather than from its contribution to global energy alone (Gaver et al., 2025). Consistent with the idea of injurious temporal focusing, waveform-level control strategies have been proposed that explicitly target the shape of intracycle power (Marini et al., 2021; Darowski et al., 2026). These translational directions would require explicit assumptions (e.g., mapping impedance changes to volume and separating resistive from elastic contributions) and prospective validation against regional stress/strain surrogates.

Therefore, we frame PI as a direction for heterogeneity-aware interpretation of MP rather than an immediately deployable clinical tool. Future model extensions should incorporate multi-compartment heterogeneity distributions, recruitment/derecruitment, and more realistic expiratory behavior to better link global signals to regionally distributed injury risk. Translational work in congenital diaphragmatic hernia suggests that even simple, physiologically interpretable heterogeneity indices—such as time-constant imbalance—may help bridge mechanistic models and bedside decisions on pressure targets during conventional mechanical ventilation (Stankiewicz et al., 2022; Stankiewicz et al., 2025).

This study has several limitations that are important when interpreting PI and MP_total_ in heterogeneous lungs:

- The compartmental energy metric represents incremental (above-PEEP) airway-opening delivered energy apportioned by compartmental flow (a routing proxy). It is not a direct measure of regional parenchymal stress/strain or tissue-level energy deposition.
- The model assumes linear parallel R–C behavior and omits important nonlinear and spatial phenomena (e.g., recruitment/derecruitment, nonlinear compliance, viscoelasticity, inertance, pendelluft, and chest-wall effects). Thus, the results are best viewed as mechanistic demonstrations of non-uniqueness rather than quantitative predictions for specific clinical phenotypes.
- Expiration is simplified and all reported metrics are inspiratory-only. The expiratory phase is simulated only to close the breathing cycle and establish initial conditions for the subsequent breath; therefore, the analysis does not address expiratory energy dissipation, air trapping, or dynamic hyperinflation-related effects.

To conclude, in heterogeneous lungs, global mechanical power does not uniquely determine how inspiratory energy is partitioned between lung units. The partition index (PI), defined as an airway-opening energetic routing metric, captures preferential loading toward the more compliant compartment and highlights conditions in which acceptable global mechanical power may mask substantial preferential energetic exposure. A heterogeneity-aware interpretation of mechanical power may improve risk stratification and help motivate tailored ventilation strategies in selected phenotypes.

## Data Availability

The datasets presented in this study can be found in online repositories. The names of the repository/repositories and accession number(s) can be found below: The simulation code, parameter-sweep outputs, and figure-generation scripts supporting the findings of this study are publicly available in a public repository at: https://github.com/tomaszurbankowski/PI-model-simulations. The repository includes all scripts required to reproduce the main analyses and figures.

## Appendix A. Closed-form derivation of Paw(t) during inspiration (VCV)

The derivation below applies only to the inspiratory phase of volume-controlled ventilation, during which total airway-opening flow is prescribed. PEEP is used as the baseline reference pressure; equivalently, the formulation can be written in terms of incremental airway pressure

$$\text{Δ}P_{aw}\left(\text{t}\right)=P_{aw}\left(\text{t}\right)-\text{PEEP}$$

For each compartment *i* ∈ {1, 2} in a linear parallel R–C model with shared airway-opening pressure:

$$\text{Δ}P_{aw}\left(\text{t}\right)=\frac{V_{i}\left(t\right)}{C_{i}}+R_{i}\cdot F_{i}\left(t\right)$$

or equivalently:

$$P_{aw}\left(\text{t}\right)=\text{PEEP}+\frac{V_{i}\left(t\right)}{C_{i}}+R_{i}\cdot F_{i}\left(t\right)$$

Rearranging gives the compartmental flow:

$$F_{i}\left(\text{t}\right)=\frac{P_{aw}\left(t\right)-PEEP-\frac{V_{i}\left(t\right)}{C_{i}}}{R_{i}}=\frac{\text{Δ}P_{aw}\left(t\right)-\frac{V_{i}\left(t\right)}{C_{i}}}{R_{i}}$$

Under volume-controlled inspiration, the prescribed total flow satisfies:

$$F_{tot}\left(t\right)=F_{1}\left(t\right)+\;F_{2}\left(t\right)=\sum_{i=1}^{2}\frac{P_{\left(aw\right)\left(t\right)}-\;PEEP\;-\frac{V_{i\left(t\right)}}{C_{i}}}{R_{i}}$$

Collecting terms in Paw(t):

$$F_{tot\left(t\right)}=\;\left(P_{aw\left(t\right)}-\;PEEP\right)\sum_{i=1}^{2}\frac{1}{R_{i}}-\;\sum_{i=1}^{2}\frac{V_{i\left(t\right)}}{C_{i}R_{i}}$$

Solving for Paw(t):

$$P_{aw\left(t\right)}=\;PEEP\;+\frac{F_{tot\left(t\right)}+\;\sum_{i=1}^{2}\frac{V_{i\left(t\right)}}{C_{i}R_{i}}}{\sum_{i=1}^{2}\frac{1}{R_{i}}}$$

Equivalently, in incremental form:

$$\Delta P_{aw}\left(t\right)=\frac{F_{tot}\left(t\right)+\sum_{i}\left(\frac{V_{i}\left(t\right)}{C_{i}R_{i}}\right)}{\sum_{i}\left(\frac{1}{R_{i}}\right)}$$

This expression was used during inspiration to compute Paw(t) at each time step before updating compartmental flows and volumes.
